# Isomer-Resolved Mass Spectrometry Imaging of Acidic Phospholipids

**DOI:** 10.1021/jasms.3c00192

**Published:** 2023-08-15

**Authors:** Britt S. R. Claes, Andrew P. Bowman, Berwyck L. J. Poad, Ron M. A. Heeren, Stephen J. Blanksby, Shane R. Ellis

**Affiliations:** † The Maastricht MultiModal Molecular Imaging (M4I) institute, Division of Imaging Mass Spectrometry (IMS), Maastricht University, 6229 ER Maastricht, The Netherlands; ‡ Central Analytical Research Facility, Queensland University of Technology, Brisbane, Queensland 4000, Australia; § School of Chemistry and Physics, Queensland University of Technology, Brisbane, Queensland 4000, Australia; ∥ Molecular Horizons and School of Chemistry and Molecular Bioscience, University of Wollongong, Wollongong, New South Wales 2522, Australia

## Abstract

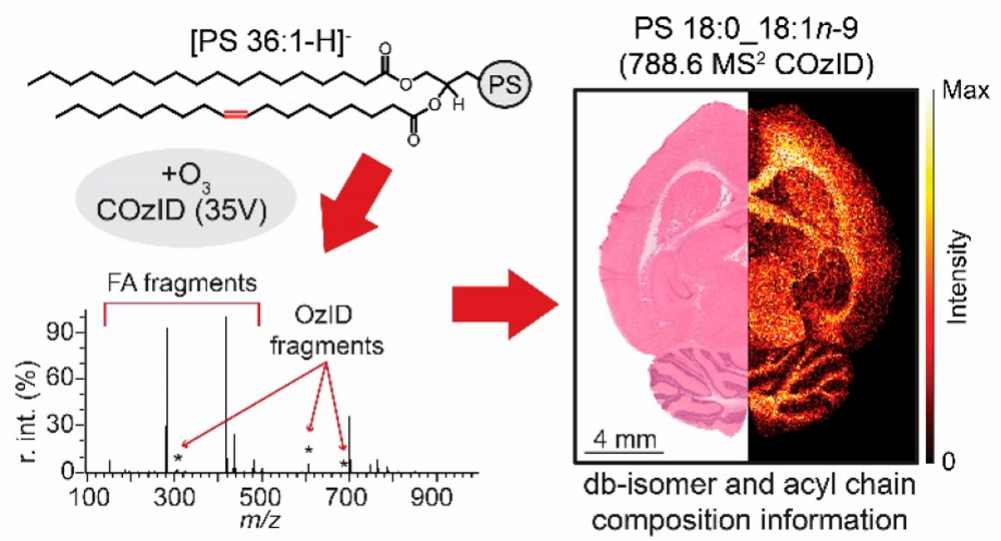

The biological functions of lipids are entirely dependent on their molecular structures with even small changes in structure—such as different sites of unsaturation—providing critical markers for changes in the underlying metabolism. Conventional mass spectrometry imaging (MSI) approaches, however, face the twin challenges of mixture and structural complexity and are typically unable to differentiate lipid isomers that differ only in the position(s) of carbon–carbon double bonds. Recent coupling of ozone-induced dissociation (OzID) with matrix-assisted laser desorption/ionization (MALDI)-MSI has demonstrated the potential to map changes in individual double-bond isomers, thus enabling visualization of the modulation in lipid desaturation in adjacent tissue types. This has, to date, only been performed in positive-ion mode due to a generally higher abundance of phosphatidylcholines (PC) in mammalian tissues and the efficient desorption/ionization of this lipid subclass. Many other glycerophospholipids (GPLs), however, are better detected in negative-ion mode as deprotonated anions. Recently, OzID has been implemented on a traveling-wave ion-mobility mass spectrometer (Waters, SYNAPT G2-S*i*) that provides a 50-fold increase in the rate of the gas-phase reaction between ionized lipids and ozone and a commensurate increase in sensitivity for isomer-resolved mass spectrometry. These gains are exploited here to interrogate the distributions of anionic GPL isomers in biological tissues, covering the subclasses phosphatidylserine (PS), phosphatidylethanolamine (PE), phosphatidylinositol (PI), phosphatidylglycerol (PG), and phosphatidic acid (PA). Exploiting both ozone- and collision-induced dissociation in a single acquisition simultaneously identifies sites of unsaturation and acyl chain composition from the same mass spectrum.

## Introduction

The identification of biomarkers in assessing both the health of tissue and the progress of disease is one of the key challenges facing clinicians. Within that context, lipidomics is an important field of study, since lipids are both major building blocks for cellular structures^1^ and critical signaling molecules.^2^ Furthermore, lipids have shown to be key players in various biological processes, such as apoptosis^3^ or altered metabolism in cancer.^4^ It is well-known that the biochemical and biophysical properties of lipids are critically dependent on their chemical structures, with relatively minor structural changes often promoting sweeping physiological differences.^5−10^ Add to this evidence for changes in lipid synthesis being dependent upon tissue type,^11^ cancer metabolism,^4,12^ and metabolic disorders (such as diabetes),^13^ and the need for unambiguous lipid identification becomes apparent.

One of the main challenges in conventional mass-spectrometry-based lipidomics lies in differentiating lipids that have very similar masses (i.e., isobars) or the exact same mass but with different molecular structures (i.e., isomers).^14−16^ These twin challenges result in lipids being typically annotated as a composite of multiple possible lipids rather than as a singular biomolecule. This problem is compounded within mass spectrometry imaging (MSI), where the spatiotemporal nature of the acquisition prohibits the inclusion of chromatographic fractionation, which could distinguish between isobars or isomers. To address this challenge, different MSI technologies have been advanced. High-resolution mass spectrometers can be used to improve isobaric resolution, with the most powerful Fourier-transform instruments providing subparts-per-million mass accuracy and resolving powers exceeding 10^6^.^17^ While this has been a significant achievement, lipid isomers cannot be resolved by mass. Ion mobility has been demonstrated to resolve some types of lipid isomers prior to mass analysis and is compatible, in some configurations, with MSI workflows.^17−19^ Many lipid isomers, however, can only be resolved by ultrahigh-resolution ion mobility with a consequent loss of duty cycle, while the assignment of mobility-resolved features to individual lipid molecular species requires careful reference to libraries^20^ of collision-cross sections or integration with selective ion activation modalities.

MSI experiments to identify the specific localization of resolved and identifiable lipid isomers have been pushed forward by a number of different ion activation methodologies.^21^ Being available on most commercial instruments, the most common form of MS/MS has been low-energy collision-induced dissociation (CID). CID mass spectra can be used to confirm the lipid class (or subclass), as well as the total number of carbons and the degree of unsaturation: a so-called sum composition assignment. For glycerophospholipids (GPLs) CID mass spectra in positive-ion mode typically yield fragmentation diagnostic of the polar headgroup, while negative-ion CID mass spectra carry abundant product ions that identify the number of carbons and degree of unsaturation in the associated acyl chains. Conventional CID mass spectra of GPLs in either polarity do not provide information relating to either the position(s) of carbon–carbon double bonds (dbs) nor the relative positions of acyl chains on the glycerol backbone (*sn*-isomers). Next-generation ion activation technologies have emerged specifically to address these limitations and enable isomer resolution. For MSI, identification of db-position(s) in GPLs can be broadly divided into two categories: (1) on-tissue (or post-tissue) derivatization methods and (2) new ion activation methods inside the mass spectrometer in the gas phase. The first approach includes on-tissue derivatization by Paternò–Büchi (PB) reactions^22^ and oxidation during nano-DESI,^23^ followed by CID of the derivatives to yield double bond specific product ions. Application of these approaches in MSI has been successful in visualizing the distribution of different db-isomers, but the treatment of the sample prior to analysis impacts the desorption/ionization efficiency and increases overall mixture complexity. The second approach leverages recent developments in ion-activation modalities to structurally interrogate ions and has been demonstrated using ultraviolet photodissociation (UVPD),^24^ ion–ion reactions,^25,26^ and ozone-induced dissociation (OzID).^27^ To date, these ion activation modalities have only been demonstrated for the MSI of phosphatidylcholine (PC) ions in the positive-ion mode.

In an MSI context, OzID has previously been applied to alkali-adducted PC lipids in positive ion mode due to both the enhanced reaction rate of alkali-adducted species with ozone^28^ and their high abundance in tissue samples.^29^ Many important GPL subclasses are more readily ionized in negative-ion mode as deprotonated anions (and cannot be observed in positive-ion mode), but the often lower reaction rate of deprotonated unsaturated lipids toward ozone has thus far impeded the use of OzID in negative mode for imaging applications. Recently, we reported the implementation of high-pressure OzID exploiting the greater number density afforded in the ion mobility region of a SYNAPT G2-S*i* system mass spectrometer for ozonolysis.^30^ This approach resulted in a 1000-fold increase in OzID reaction rates that significantly enhanced the capabilities of OzID-MSI.

Herein, we demonstrate the application of high-pressure OzID to isomer-resolved imaging of several classes of deprotonated phospholipids, including phosphatidylserine (PS), phosphatidylethanolamine (PE), phosphatidylinositol (PI), phosphatidylglycerol (PG), and phosphatidic acid (PA). Combining CID with OzID in a single concerted collision-ozone-induced dissociation (COzID) method enabled simultaneous identification of acyl chain and db-position. We demonstrate the utility of this combined approach using healthy rat brain tissue as an example as well as its application to diseased tissue using medulloblastoma-bearing mouse brain tissue.

## Methods

### Chemicals

Norharmane and chloroform (≥99%) were purchased from Sigma-Aldrich (Zwijndrecht, The Netherlands) and used without further purification. Methanol (ULC-MS grade), water (LC-MS grade), ethanol (LC-MS grade), and xylene (AR grade) were purchased from Biosolve (Valkenswaard, The Netherlands). Hematoxylin and Entellan were purchased from Merck (Darmstadt, Germany), and eosin Y was purchased from J.T. Baker (Center Valley, USA). Indium tin oxide (ITO) coated glass slides were purchased from Delta Technologies (Loveland, USA). A lipid standard of PS 18:0/18:1*n*-9 was purchased from Avanti Polar Lipids (Alabaster, USA).

### Biological Samples

Rats, housed and cared for at the Central Animal Facility of Maastricht University according to local standards, were provided *ad libitum* access to water and regular chow. Healthy rat brains were obtained in accordance with protocols approved by the Animal Care and Use Committee (DEC number 2014-120). Medulloblastoma-bearing mouse brains from Transgenic ND2:SmoA1-GFP mice were housed and cared for at Emory University and used in accordance with protocols approved by the Emory Institutional Animal Care and Use Committee. Horizontal sections of all tissues measuring 10 μm thick were prepared using a cryomicrotome (Leica, Nussloch, Germany) at −20 °C, thaw-mounted on ITO-coated glass slides, and stored at −80 °C until matrix application and MSI analysis.

### Sample Preparation

An automated TM-Sprayer (HTX Technologies, LLC, Chapel Hill, USA) was used for the application of the lipid standard between 0.01 and 0.1 g/L concentrations in 2:1 CHCl_3_:MeOH onto clean ITO slides for 1 to 10 layers using the following protocol: spray flow rate 0.12 mL/min, 30 s drying time between layers, velocity of 1200 mm/min, 3 mm track spacing, at 30 °C. This created concentrations from 0.33 to 3.3 ng/mm^2^ (assuming equal dispersion). Samples were then treated equivalently to brain tissue sections, where 15 layers of 7 mg/mL norharmane matrix in 2:1 CHCl_3_:MeOH were sprayed using the TM-Sprayer using the same settings used for the lipid standards.

Hematoxylin and eosin (H&E) staining was performed after MALDI imaging. Matrix was removed from tissue by immersion in 70% ethanol for 3 min. A standard H&E protocol was then used (70% EtOH, Milli-Q, hematoxylin, running tap water for 3 min; eosin for 30 s; running tap water for 3 min; 100% EtOH for 1 min; xylene for 30 s; covered with a coverslip using Entellan mounting medium). High-resolution optical images of stained tissues were generated using an Aperio CS2 digital pathology slide scanner (Leica Biosystems, Wetzlar, Germany). Annotations of the rat brain are shown in Figure S1.

### Mass Spectrometry Imaging Instrumentation

Tissue sections were analyzed using a Waters prototype μMALDI source interfaced with a Waters SYNAPT HDMS G2-S*i* ion mobility-enabled quadrupole time-of-flight mass spectrometer (Waters Corporation, Manchester, UK).^31^ Samples were analyzed in continuous raster mode using Waters Research Enabled Software (WRENS) to operate at 5 pixels/s, a laser repetition rate of 1500 Hz, a pixel size of 50 μm, and a quadrupole set to transmit a mass window of ±1.5 Da. A reflectron time-of-flight analyzer was operated in sensitivity mode, yielding a resolving power of *m*/Δ*m* ≈ 15000. The laser spot size was approximately 15 × 15 μm. Operation of the T-Wave was optimized as previously described.^30^ For OzID, the traveling wave velocity and amplitude were 1800 m/s and 36 V, respectively. For the COzID 1000 m/s and 38 V were used for traveling wave velocity and amplitude, respectively. Optimization of the ion mobility traveling wave parameters permitted the detection of PS 36:1 down to 4.4 fg/μm^2^ (Figure S2). The trap collision energy was varied according to GPL class between 20 and 35 V for COzID, while the transfer collision energy was set to 2.0 V. Additional sections were analyzed using a MALDI-enabled Orbitrap Elite (Thermo Scientific, Bremen, Germany) at mass resolution 240000 (at *m*/*z* 400) to probe for isobaric lipid species in the lower mass resolution data sets. MS/MS was performed in the HCD cell of the Orbitrap using an isolation width of 1 Da and a normalized collision energy of 30 (manufacturing units).

### In-Line Ozone Generation

Ozone generation and delivery to the instrument were as described previously.^32^ Briefly, ozone was produced with a high-concentration ozone generator (TG-40; Ozone Solutions, Hull, IA, USA) from UHP oxygen (5.0 grade, 20 psi at 0.4 slm; Linde Gas Therapeutics Benelux bv, Eindhoven, The Netherlands). Ozone concentration in O_2_ was maintained at 280 g/m^3^ as monitored in-line using a UV-absorption-based ozone monitor (106-H; 2B Technologies, Boulder, USA). Ozone was then introduced into the ion mobility cell gas line of the mass spectrometer with the total pressure in the IMS cell maintained at 2.3 mbar. Excess ozone was destroyed using an unheated destruct catalyst (810-0008; In USA, Inc., Norwood, USA). Laboratory ambient ozone concentration was monitored using a low-concentration ozone monitor (106-L; 2B Technologies, Boulder, USA) and interlocked to shut off the generator if the background ozone level rose above 75 ppb.

### Data Analysis

WatersRawConverter (Waters Corporation, Manchester, UK) was used to convert WRENS data by using a bin size of 1 Da. Data were visualized using in-house-created MATLAB scripts (version R2014a, MathWorks, Natick, USA). Regions of interest (ROIs) were manually selected to remove off-tissue regions when plotting images. All MS^1^ images were TIC normalized, while for OzID isomer images 99th quantile hotspot removal was performed on the non-normalized images. Fractional distribution images (FDIs) were created with the numerator being the sum of the aldehyde and Criegee OzID fragments of a single isomer and the denominator being the sum of the OzID fragments for all isomers. An overview of these fragments of the lipids studied can be found in Tables S1 and S2. Mass spectra were averaged in MassLynx v4.1 and exported to mMass software for offline recalibration and peak picking (S/N = 3). Extracted ion chromatograms were obtained from MassLynx to determine individual scan noise levels to define the limit of blank and limit of detection. Calibrant peaks were the most abundant OzID product ions, along with the headgroup fragment and the ozonide of the precursor lipid. Stacked box plots were plotted in GraphPad Prism 9.3.1 using the percentages calculated from five ROIs from each tissue type, based on comparison with the H&E.

### Lipid Nomenclature

Lipid structure nomenclature is based on the recommendations of Liebisch et al.^33^ When the identity of an acyl chain is known, an underscore (_) or slash (/) is used for unknown or known *sn*-positions of acyl chains, respectively. Site(s) of unsaturation are indicated by *n*–*x*, where *x* is the number of carbons relative to the methyl terminus of the acyl chain.^20^ Polyunsaturated fatty acids (PUFAs) are denoted by the first db-position from the methyl terminus using the omega (ω) symbol.

## Results and Discussion

**Figure 1:**
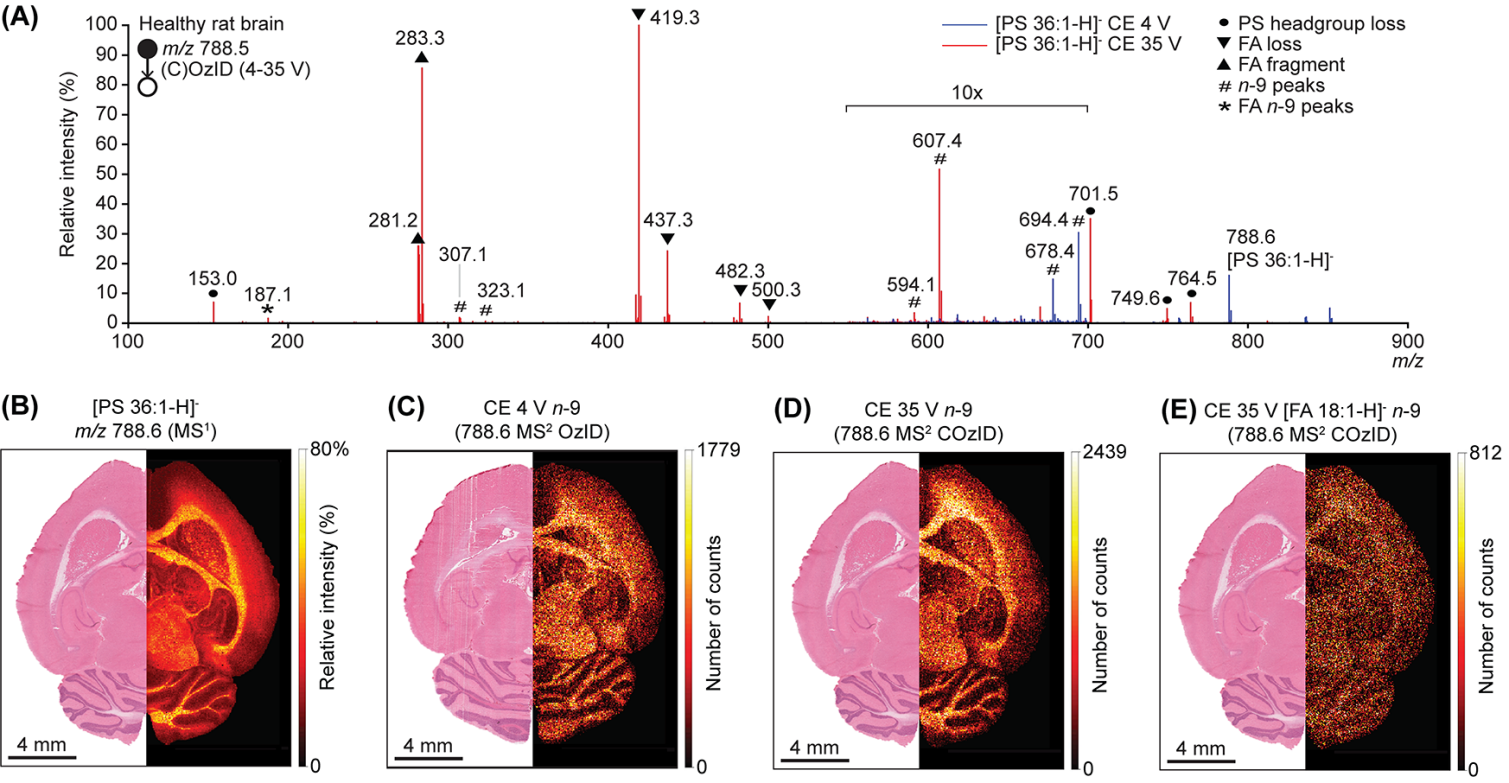
Increased collision energy results in improved sensitivity for the detection of PS 36:1. (A) Comparison of COzID spectra of [PS 36:1-H]^−^ anions acquired with trap collision energies of 4 (blue) and 35 V (red). (B) Overall distribution of [PS 36:1-H]^−^ in healthy rat brain tissue section and (C) the distribution of PS 36:1*n*-9 at low collision energy. (D) Increasing the collision energy to 35 V improved contrast in the reconstructed image by ca. 30% based on the intensities of the PS 36:1*n*-9 diagnostic product ions. (E) The increased collision energy experiment also enabled visualization of FA 18:1*n*-9 from the OzID product ions of the CID-generated 18:1 fatty acid fragment ion (*m*/*z* 281).

In positive-ion mode, monounsaturated phosphatidylcholine lipids have been previously identified as abundant species and the spatial distributions of different sites of unsaturation were found to be localized in discrete sections of the tissue.^27,30,34^ Following the same logic, several monounsaturated lipids from acidic phospholipid classes were chosen for investigation in the rat brain. Sections of normal rat brain were ionized using MALDI in negative mode and produced an abundant signal at *m*/*z* 788.5, assigned as deprotonated [PS 36:1-H]^−^, with a spatial distribution that could be coregistered with tissue features in the H&E stain. Mass selection of [PS 36:1-H]^−^ and subsequent ion activation using combinations of CID and OzID allowed for the structural interrogation of the lipid. Systematically increasing the collision energy (CE) in the trap region of the instrument prior to introduction of the ions to the traveling-wave ion-mobility spectrometry (TWIMS) cell produced abundant CID product ions, in addition to enhancing the abundance of the OzID product ions (Figure 1A). Imaging deprotonated [PS 36:1-H]^−^ at the MS^1^ level showed an increased abundance of the intact lipid ion in the white matter of healthy rat brain tissue compared to the gray matter (Figure 1B). At a trap CE of 4 V, OzID product ions attributable to PS 36:1*n*-9 db isomer were observed, following the same white/gray matter distributions as the precursor ion in MS^1^ (Figure 1C). When the CE was raised to 35 V, diagnostic product ion intensities for both CID and OzID products were increased between 2- and 100-fold (Figure 1D), identifying the presence of 18:0 and 18:1 fatty acyl chains (Figure 1A). Although information on the *sn*-position via CID/OzID is not available in negative mode, as is the case from alkali adducted species in positive mode, the improved OzID sensitivity permitted imaging of the *n*-9 db isomer from the CID-generated fatty acid (FA) 18:1 product ion, showing that it follows the same spatial distribution as the PS 36:1*n*-9 isomer imaged from the intact precursor (Figure 1E). Critically, this synergistic combination of ion activation strategies demonstrates the capability of imaging db isomers specific for individual fatty acids, presenting complementary information to either technique on their own.

The extra sensitivity afforded by the COzID method identified that PS 36:1 is comprised of 2 acyl chain isomers, PS 16:0_20:1 and PS 18:0_18:1, with PS 18:0_18:1 being the dominant isomer in the rat brain (>98% of the total signal) (Figure S3). Clear differences in the spatial distributions of the acyl chain isomers are observed, where PS 18:0_18:1 followed a trend similar to that of the intact PS 36:1*n*-9 precursor distribution, while PS 16:0_20:1 followed a more homogeneous distribution across the rat brain, albeit at low signal-to-noise. Interestingly, only OzID product ions consistent with PS 36:1*n*-9 were detected. This contrasts with our previous positive ion mode imaging work where both PC 36:1*n*-7 and PC 36:1*n*-9 db isomers were identified.^27,30^ Both PS 36:1 and PC 36:1 show the same MS^1^ overall distribution, and previous studies have demonstrated that PC 36:1*n*-9 was significantly more abundant in white matter in rat brain.^27^

**Figure 2:**
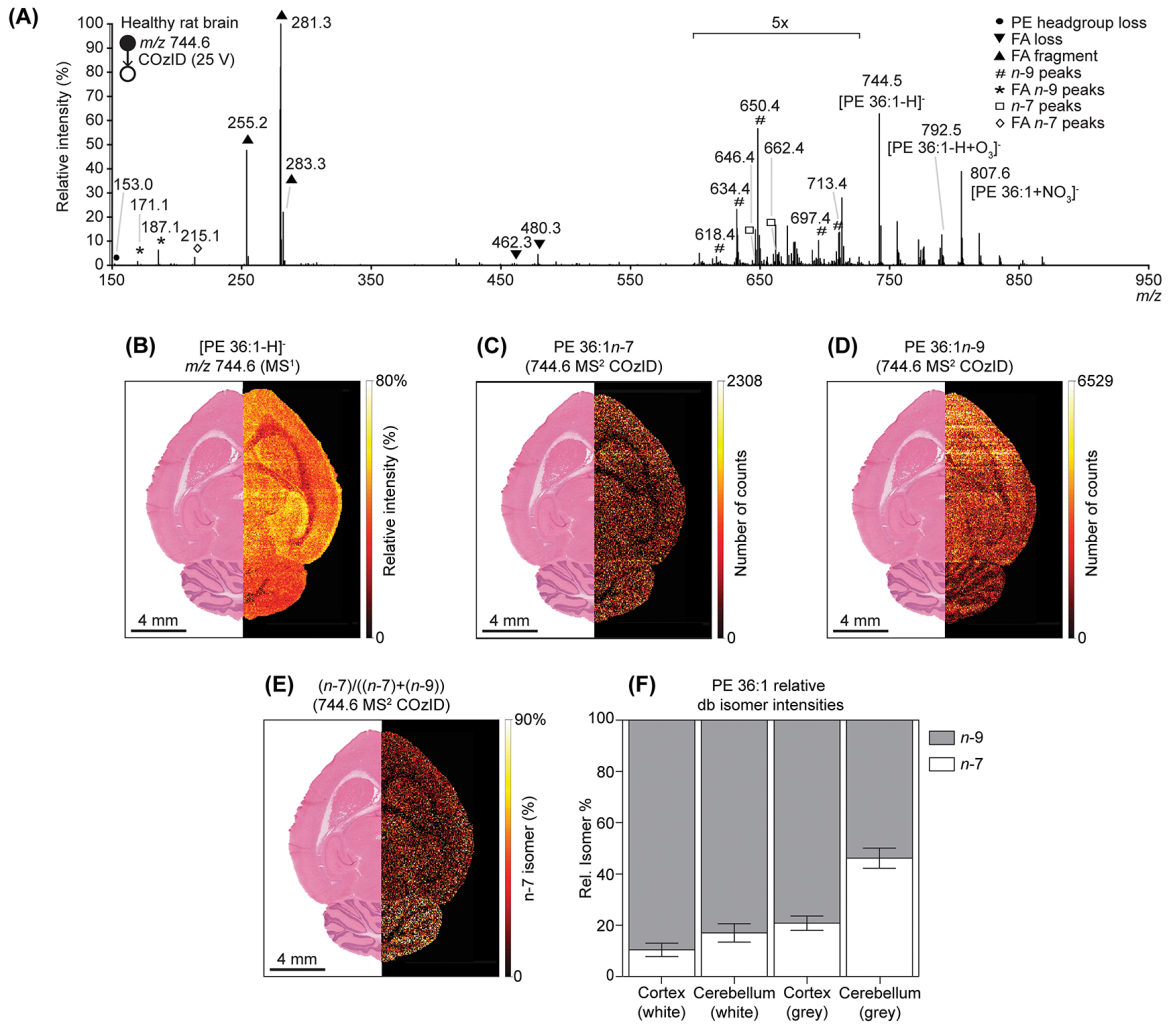
Change in the db positions in [PE 36:1-H]^−^. (A) COzID spectrum of PE 36:1 showing evidence for both OzID and CID product ions permitting structural elucidation. (B) Total signal for [PE 36:1-H]^−^ in the rat brain highlighted generally in the gray matter compared to the H&E. (C) The PE 36:1*n*-7 isomer appears upregulated in gray matter and is less abundant in white matter. (D) In contrast, the PE 36:1*n*-9 isomer is more abundant and appears to highlight the molecular layer in the cerebellum. (E) The FDI shows an increase of PE 36:1*n*-7 in the cerebellum but a decrease in the granular cell layer relative to PE 36:1*n*-9. (F) The box plot with relative db isomer intensities shows an increase of 2- to 3-fold of the PE 36:1*n*-7 isomer in the gray matter of the cerebellum compared to the white matter and the cortex of the brain. Error bars show the standard deviations of the ROIs.

Mass selection and subsequent COzID-MSI of [PE 36:1-H]^−^ (*m*/*z* 744.55) showed the presence of both PE 36:1*n*-7 and PE 36:1*n*-9 isomers, with PE 36:1*n*-9 being more abundant (Figure 2A). Despite the diagnostic information being spread across multiple adducts and product ion channels, these could be treated together to reconstruct the spatial distributions of each isomer. The FDIs for PE 36:1*n-*7- and PE 36:1*n*-9-related product ions are shown in Figure 2, generated by using the combined ion signals of all isomer-specific fragments. As the ionization efficiencies of phospholipids are determined by the headgroup, it is expected that each db isomer ionizes to the same extent and thus changing isomer ratios across the tissue reflect an underlying change in the abundance of each isomer across the tissue. At the MS^1^ level, the distribution of [PE 36:1-H]^−^ (representing the sum of all isomers) showed a higher abundance in gray matter compared to white matter (Figure 2B). The PE 36:1*n*-7 isomer appeared upregulated in the gray matter (Figure 2C), while PE 36:1*n*-9 was more abundant in the granular layer in the cerebellum and the gray matter in the cortex (Figure 2D). The FDI showed an increase of PE 36:1*n*-7 isomer proportion in the cerebellum but a decrease in the granular layer (Figure 2E). The box plot of relative signal intensities for the isomers also clearly shows the increase of PE 36:1*n*-7 in the gray matter of the cerebellum, appearing 2- to 3-fold higher compared to white matter and the cortex (Figure 2F). From the COzID data, the fatty acyl compositions for PE 36:1 were putatively assigned as PE 18:0_18:1 and PE 16:0_20:1, similar to the acyl chain compositions observed for PS 36:1. However, the OzID product ions from the CID generated fatty acyl anions were of too low abundance to construct a visualization and assign db-location to individual fatty acyls.

**Figure 3:**
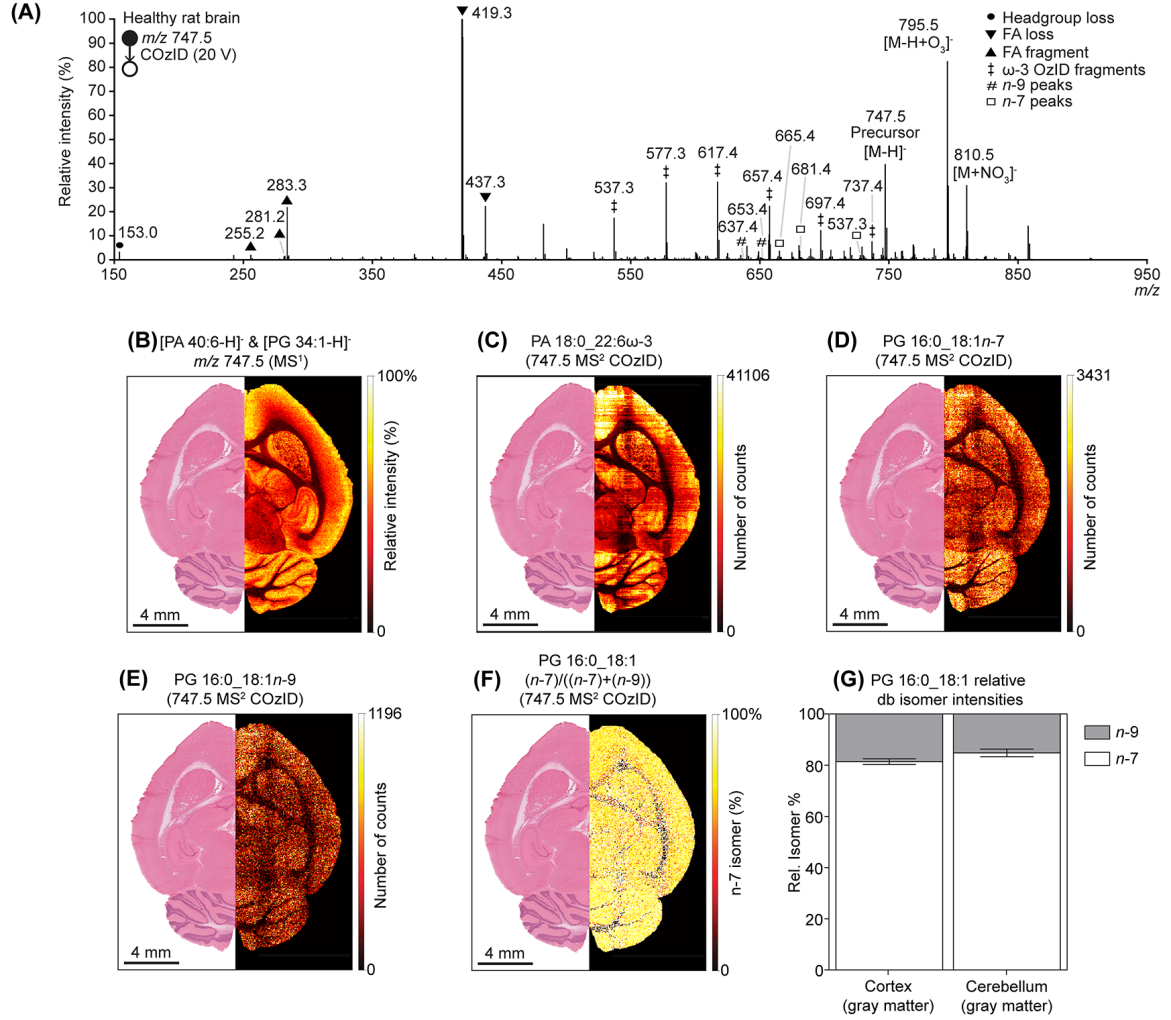
Differentiating between isobaric and isomeric lipids simultaneously using COzID. (A) COzID spectrum of *m*/*z* 747.5, displaying overlaid product ions from both [PA 40:6-H]^−^ and [PG 34:1-H]^−^. (B) Unresolved imaging of [PA 40:6-H]^−^ and [PG 34:1-H]^−^ shows depletion in the white matter of the brain, while highlighting the molecular layer of the cerebellum and the dentate gyrus. (C) The spatial distribution of PA 18:0_22:6ω-3 shows a similar increase, highlighting the molecular layer of the cerebellum as well as the gray matter of both the cerebellum and cortex. (D) Imaging of PG 16:0_18:1*n*-7 highlights the gray matter of the brain, with a greater abundance in the cerebellum. (E) The PG 16:0_18:1*n*-9 isomer shows no highlight except for depletion of the white matter of the brain. (F) The FDI of PG 16:0_18:1 shows a homogeneous abundance of *n*-7 in the gray matter of the brain. (G) Highlighting the very high proportion of *n*-7 relative to *n*-9 isomer populations in PG 16:0_18:1 with no changes in this fraction noted between the cortex and cerebellum regions. Error bars show the standard deviation of the ROIs.

COzID also proved to be beneficial in resolving isobaric lipids. For example, both [PA 40:6-H]^−^ (*m*/*z* 747.4970) and [PG 34:1-H]^−^ (*m*/*z* 747.5182) are often detected in brain tissue but differ by only 21 mDa. While the resolving power required to distinguish these isobars (*m*/Δ*m* ≈ 35000) exceeded that afforded by the present instrument, they are readily distinguished using COzID based on the acyl chain composition while still identifying db isomers belonging to each lipid (Figure 3A). The presence of both lipids in the rat brain was verified using high-resolution/accurate mass and tandem MS (on a Thermo Orbitrap Elite; Figure S4). For PA 40:6, only a single db isomer was detected, assigned as PA 18:0_22:6ω-3. For PG 34:1 both monounsaturated fatty acids (MUFA) with *n*-7 and *n*-9 were detected. Imaging of the MS^1^ ion showed slight increases in the molecular layer of the cerebellum in the rat brain as well as within the dentate gyrus (Figure 3B). Imaging of the PA 18:0_22:6ω-3 fragments showed a similar trend, highlighting the molecular layer of the cerebellum and the dentate gyrus as well as the gray matter of the brain as a whole (Figure 3C), consistent with previous findings.

Investigation of PG 16:0_18:1*n*-7 in the healthy rat brain showed morphology similar to that of the MS^1^ ion image, with abundance in the gray matter and a higher intensity in the cerebellum compared to the cortex of the brain (Figure 3D). The lower abundance PG 16:0_18:1*n*-9 isomer showed no particularly distinct features except that it was not found in white matter. This latter trend is similar to the combined image of the [PA 40:6-H]^−^ and [PG 34:1-H]^−^ precursor ions (Figure 3B,E). The FDI showed a consistently higher intensity of *n*-7 signals compared with the *n*-9 isomer throughout the gray matter of the brain (Figure 3F), with the cortex and cerebellum reporting up to 80% of the PG 34:1 signals attributed to PG 34:1*n*-7 (Figure 3G). The greater abundance of PG 16:0_18:1*n*-7 compared to PG 16:0_18:1*n*-9 contrasts with what was found for the PS 36:1 and PE 36:1 discussed above. Similar db isomers were found previously for PC 34:1 in healthy rat brain, where PC 34:1*n*-7 was found to be proportionately more abundant in the gray matter of the cerebellum.^27,30^ Interestingly, while PG 34:1 was found to be comprised of 80% *n*-7, PC 34:1*n*-7 was not found to be greater than 40%.^27^ This contrast between PG and PC highlights the need for a class-specific mapping of unsaturation.

In general, the PUFA GPLs surveyed here in negative-ion mode were found to exist as only one double bond isomer at the sum composition level (Figure S5), consistent with previous findings for PC lipid species in positive mode.^27,30^ For example, the OzID of mass-selected PE 38:4 (*m*/*z* 766.6; Figure S5A) produced product ions diagnostic for ω-6 unsaturation. Combining this information with the CID product ion at *m*/*z* 303.5 identified the PUFA acyl chain as arachidonic acid and enabled the assignment of the precursor lipid as PE 18:0_20:4ω-6. This finding is consistent with that of Bednarik et al.,^35^ where the same double bond positions were identified using a PB-MALDI-2-MS/MS approach that required prior on-tissue derivatization. The most abundant ions for [PE 38:4-H]^−^ were found within the dentate gyrus, ventricle lining, and molecular layer of the cerebellum in the rat brain. OzID product ions specific for FA 20:4ω-6 were also found within the same tissue region, supporting our putative assignment. Similarly, [PI 38:4-H]^−^ was observed as a single db-isomer, PI 18:0_20:4ω-6 (Figure S5B). Healthy rat brain showed the largest abundance of [PI 38:4-H]^−^ in gray matter; however, the cerebellum, the dentate gyrus, and the ventricle lining seem to be more broadly enhanced with PI 18:0_20:4ω-6, rather than distinctly focused on the molecular layer as in PE 18:0_20:4ω-6.

**Figure 4:**
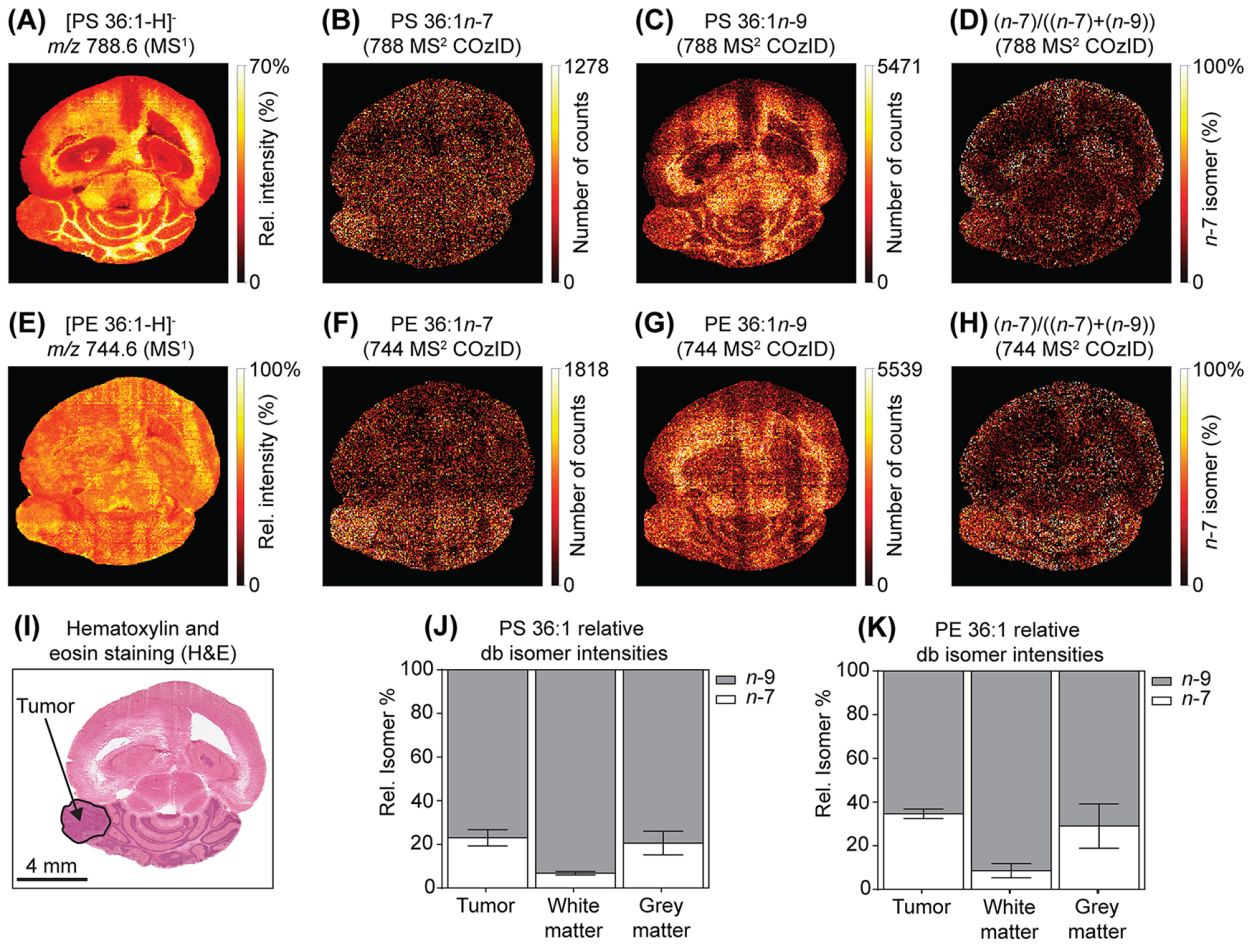
Imaging of db isomers of PS 36:1 and PE 36:1 in mouse brain-bearing medulloblastoma. (A) MS^1^ imaging of [PS 36:1-H]^−^ showed an increased presence in white matter and decreased presence in the tumor tissue. (B) In contrast to healthy rat brain, an *n*-7 db isomer of PS 36:1 was detected in the mouse brain and was upregulated in the tumor region. (C) The *n*-9 db isomer of PS 36:1 followed the primary ion distribution, being upregulated in the white matter. (D) FDI of *n*-7 over *n*-7 + *n*-9 showed PS36:1*n*-7 increase in the gray matter and tumor tissue. (E) [PE 36:1-H]^−^ showed a uniform signal, being only slightly decreased in the ventricle. (F) In contrast to the rat brain, the *n*-7 isomer of PE 36:1 was shown to be more abundant in the cerebellum of the mouse, while (G) the *n*-9 isomer of PE 36:1 was shown to be more abundant in the white matter. (H) The FDI of PE 36:1 showed an increase of the PC 36:1*n*-7 isomer in the tumor region and the gray matter. (I) H&E of the mouse brain shows the tumor on the left of the cerebellum. Box plots of the relative db isomer intensities of (J) PS 36:1 and (K) PE 36:1 in the tumor region, white matter, and gray matter. Error bars show the standard deviation of the ROIs.

To demonstrate the utility of COzID for imaging aberrant metabolism, imaging experiments were conducted on mouse brains bearing medulloblastoma. The CID product ions detected from PS 36:1 and PE 36:1 showed that both lipids contained the same fatty acyl compositions as detected in the healthy rat brain: i.e., PS 18:0_18:1, PS 16:0_20:1, PE 18:0_18:1, and PE 16:0_20:1. Furthermore, these mouse brains showed [PS 36:1-H]^−^ distributions that followed the same pattern as that in healthy rat brain tissue, being highlighted in the white matter (Figure 4A,I). However, the PS 36:1*n*-7 isomer was detected in the mouse brain and was slightly more abundant in the medulloblastoma and gray matter (Figure 4B), while PS36:1*n*-9 followed the same spatial distribution as the precursor (Figure 4C). The *n*-7 FDI highlighted both the gray matter and the tumor in the cerebellum (Figure 4D), indicating an upregulation of PS 36:1*n*-7, which correlates with the PS 36:1*n*-7 distribution. While the medulloblastoma-bearing mouse brain showed no specific distribution for the precursor PE 36:1 (Figure 4E,I), differences in the double bond isomer distributions were noted. The PE 36:1*n*-7 isomer was more abundant in the cerebellum of the mouse and the tumor region (Figure 4F), while PE 36:1*n*-9 was more abundant in the white matter (Figure 4G). The FDI showed an upregulation of PE 36:1*n*-7 in both the tumor region and the gray matter of the cerebellum (Figure 4H). Both trends in the FDI are in good agreement with that reported previously for PC 36:1,^27,30^ indicating that the metabolic pathways active in the tumor region can lead to the synthesis of PS and PE lipids containing unusual sites of saturation. Comparing the isomer proportions for both PS 36:1 (Figure 4J) and PE 36:1 (Figure 4K) showed an overall similar abundance of the respective *n*-7 isomers throughout the tumor and gray matter of the brain, with a slightly lower proportion in white matter.

This pattern of increase in the proportion of the *n*-7 isomer was consistent across all of the monounsaturated GPLs studied here; PE 36:1, PG 34:1, and PS 36:1 all display an increased proportion of *n*-7 versus *n*-9 in the cerebellum. This agrees with previous positive-ion imaging studies, which showed the same trend for PC 34:1 and PC 36:1.^27,30^ The relative accumulation of *n*-7 isomers is possibly a result of the relatively low expression of elongation enzymes such as ELOVL6 in the cerebellum compared to the cerebral cortex or the white matter.^36^ Similar changes in ELOVL expression in glioblastoma tumors have also been described.^37^

## Conclusion

By exploiting the higher reaction rates enabled by high-pressure OzID, this work demonstrates the first application of OzID-MSI to deprotonated lipids and significantly expands the phospholipid classes that can be analyzed by OzID-MSI. Negative mode OzID-MSI revealed consistent trends across several GPL classes, in good agreement with previous work,^27,30^ with no apparent variation in the PUFA GPLs. Extension of COzID imaging to the negative-ion mode allows for accessing new information and comparing these structural differences across multiple lipid classes. Interestingly, we found similar trends between the different GPLs, such as similar fatty acyl compositions and similar sites of FA 18:1 unsaturation over multiple classes, with exception of PS 18:0_18:1*n*-7 in rat brain.

The tissues used here show the applicability of both of these techniques to multiple imaging experiments in both healthy and diseased tissue. Specific tissue types can be identified through their db-positional isomers in multiple GPL classes, and changes between these lipid classes can be identified. Tracking these changes across multiple GPL classes has thus far presented a challenge for positive-mode OzID-MSI experiments due to the poor ionization efficiencies of many acidic lipid classes in positive-ion mode.

While the explicit assignment of the *sn*-position via CID/OzID is not accessible in negative mode, it is appealing to consider a combined workflow exploiting both CID/OzID in positive-ion mode with COzID in negative-ion mode to interrogate db-positional isomers, acyl chain length variations, and connectivity to the glycerol backbone for complete structural elucidation. Such hybrid workflows will be essential to reach true molecular resolution in MSI, which may offer insight into region-specific lipid metabolism within tissues and how these are altered by disease.

## Supporting Information Available

H&E annotations of the rat brain, sensitivity test to find the limit of detection, OzID-related *m*/*z* values of the monounsaturated lipids, OzID-related *m*/*z* values of the polyunsaturated lipids, acyl chain isomers of PS 36:1 in rat brain, confirmation of the presence of both PG 34:1 and PA 40:6 in rat brain, and imaging of PE 38:4 and PI 38:4 showing low isomerization of db positions (PDF)
