# Supplementary material for: Isomer-Resolved Mass Spectrometry Imaging of Acidic Phospholipids
Source: J Am Soc Mass Spectrom. 2023 Aug 15;34(10):2269–77. doi: 10.1021/jasms.3c00192 (PMC10557375; doi:10.1021/jasms.3c00192)
Supplement: Supplementary file 1 — js3c00192_si_001.pdf [file js3c00192_si_001.pdf]

Supporting information:

Isomer-resolved mass spectrometry imaging of acidic phospholipids

Britt S.R. Claes[1#], Andrew P. Bowman[1#], Berwyck L.J. Poad[2,3], Ron M.A. Heeren[1]*, Stephen J. Blanksby[2,3]* and Shane R. Ellis[1,4,5]*

[1]The Maastricht MultiModal Molecular Imaging (M4I) institute, Division of Imaging Mass Spectrometry (IMS), Maastricht University, 6229 ER Maastricht, The Netherlands
[2]Central Analytical Research Facility, Queensland University of Technology, Brisbane, Queensland 4000, Australia
[3]School of Chemistry and Physics, Queensland University of Technology, Brisbane, Queensland 4000, Australia
[4]Molecular Horizons and School of Chemistry and Molecular Bioscience, University of Wollongong, Wollongong, New South Wales 2522, Australia

[#] These authors have equally contributed

* Corresponding authors:
Dr. Shane R. Ellis
Email: sellis@uow.edu.au
Prof. Stephen J. Blanksby
Email: stephen.blanksby@qut.edu.au
Prof. Ron M.A. Heeren
Email: r.heeren@maastrichtuniversity.nl

Table of Contents

Figure S1: Annotated H&E of the rat brain. S2
Figure S2: Sensitivity test to find the limit of detection. S2
Table S1: OzID-related *m/z* values of the monounsaturated lipids S3
Table S2: OzID-related *m/z* values of the polyunsaturated lipids S3
Figure S3: Acyl chain isomers of PS 36:1 in rat brain. S4
Figure S4: Confirmation of the presence of both PA 40:6 and PG 34:1 in rat brain S5
Figure S5: Imaging of [PE 38:4-H]- and [PI 38:4-H]- in rat brain....................S6

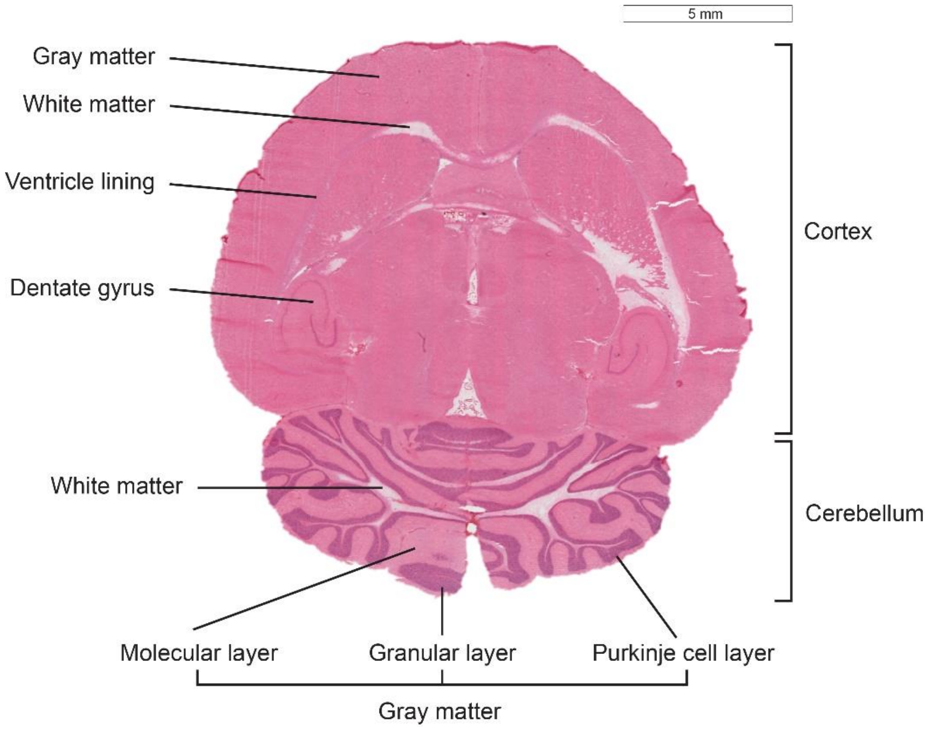

Figure S1: Annotated H&E of the rat brain.

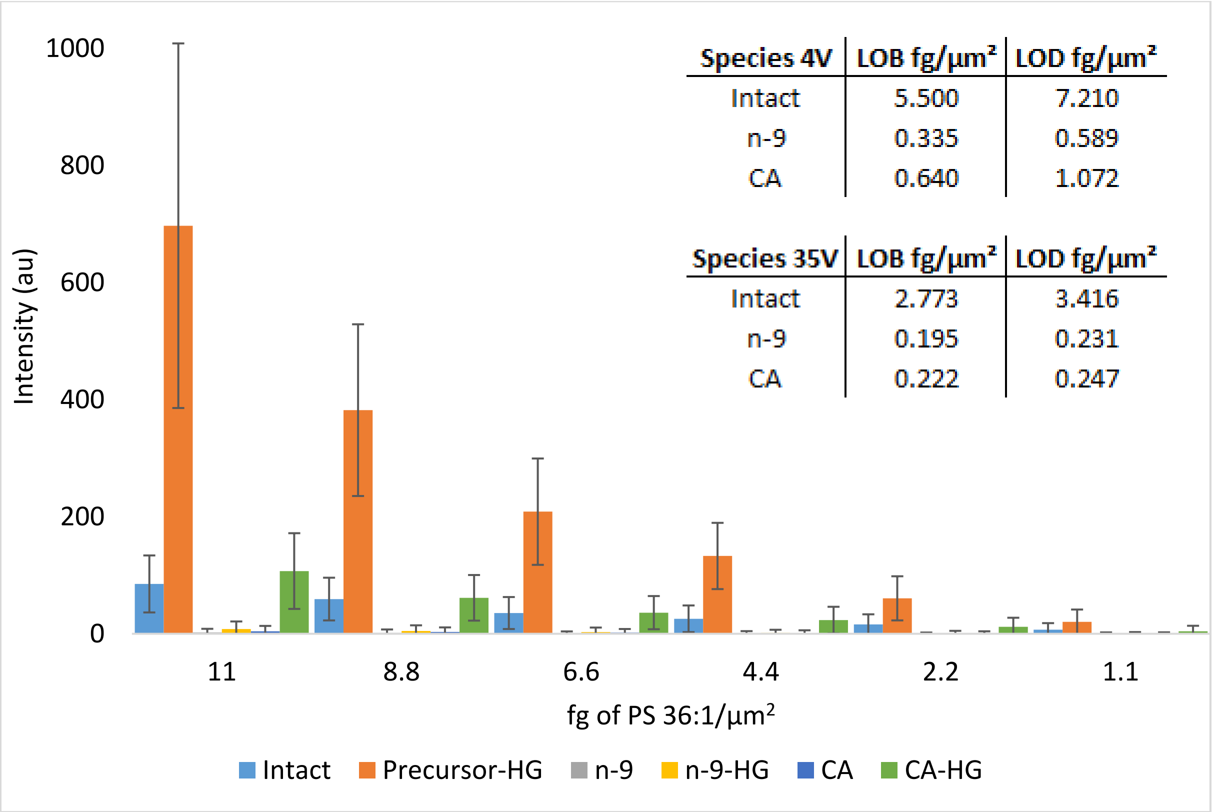

Species 4V	LOB fg/µm²	LOD fg/µm²
Intact	5.500	7.210
n-9	0.335	0.589
CA	0.640	1.072

Species 35V	LOB fg/µm²	LOD fg/µm²
Intact	2.773	3.416
n-9	0.195	0.231
CA	0.222	0.247

Figure S2: Sensitivity test to find the limit of detection. 1-10 layers of a PS 18:0/18:1*n*-9 standard were sprayed onto an ITO slide and measured by MALDI-(C)OzID under the same conditions as the brain tissue sections. To examine the sensitivity, the abundance of the precursor and OzID product ions (the *n*-9 aldehyde and Criegee ions at *m/z* 678.4 and 694.4, respectively) were monitored, including the *n*-9 aldehyde and Criegee -PS headgroup ions (*m/z* 591.4 and *m/z* 607.4, respectively). Figure values are based on ~850 individual pixels acquired from each concentration at 35 V. Characteristic OzID product ions and precursor signal for PS were observed in surface concentrations as low as 4.4 fg/µm^2.

Table S1: OzID-related *m/z* values of the monounsaturated lipids PS 36:1, PE 36:1, and PG 34:1. These include adducts with $+O_3$ and $+HNO_3$, COzID fragments, and OzID fragments -PS(87). The primary OzID fragments are shown in bold.

**Monounsaturated lipids**					
**PS 36:1**		**PE 36:1**		**PG 34:1**	
***n*-7 (*m/z*)**	***n*-9 (*m/z*)**	***n*-7 (*m/z*)**	***n*-9 (*m/z*)**	***n*-7 (*m/z*)**	***n*-9 (*m/z*)**
199.1	171.1	199.1	171.1	199.1	171.1
215.1	187.1	215.1	187.1	215.1	187.1
335.1	307.1	646.4	618.4	**665.4**	**637.4**
351.1	323.1	**662.4**	**634.4**	**681.4**	**653.4**
619.4	591.4	**678.4**	**650.4**	728.4	700.4
635.4	607.4	725.4	697.4	744.4	716.4
698.4	670.4	741.4	713.4		
**706.4**	**678.4**				
**722.4**	**694.4**				

Table S2: OzID-related *m/z* values of the polyunsaturated lipids PE 38:4, PI 38:4, and PA 40:6. These include adducts with $+O_3$ and $+HNO_3$. The primary OzID fragments are shown in bold.

**Polyunsaturated lipids**					
**PE 38:4**		**PI 38:4**		**PA 40:6**	
***n*-6**	**698.4**	***n*-6**	**817.4**	***n*-3**	**721.4**
	**714.4**		**833.4**		**737.4**
	762.4		896.4		769.4
***n*-9**	**658.4**	***n*-9**	**777.4**		800.4
	**674.4**		**793.4**	***n*-6**	**681.4**
	722.4		856.4		**697.4**
	737.4	***n*-12**	**737.4**		729.4
***n*-12**	**618.4**		**753.4**		744.4
	**634.4**		816.4		745.4
	682.4	***n*-15**	**697.4**	***n*-9**	**641.4**
	697.4		**713.4**		**657.4**
***n*-15**	**578.4**		776.4		689.4
	**594.4**				704.4
	657.3				705.4
					720.4
				***n*-12**	**601.4**
					**617.4**
					664.3
					680.3
				***n*-15**	**561.4**
					**577.4**
					624.3
					640.3
				***n*-18**	**521.4**
					**537.4**
					584.3
					600.3

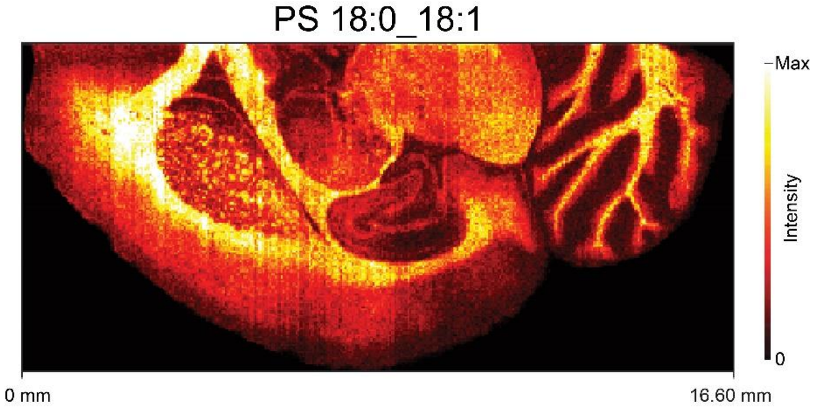

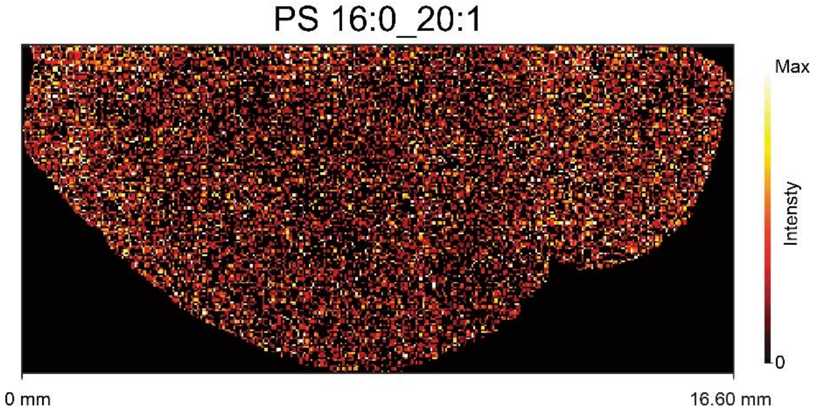

Figure S3: Acyl chain isomers of PS 36:1 in rat brain. Aside from identifying the double bond position of [PS 36:1-H]$^-$, we could determine simultaneously that PS 36:1 is mainly formed of two acyl chain isomers: 18:0_18:1 (*m/*z 281.2 and 283.3, respectively) and 16:0_20:1 (*m/z* 255.2 and 309.3, respectively), with 18:0_18:1 being the most intense acyl chain composition. PS 18:0_18:1 shows a similar trend as the *n*-9 isomer distribution and is more abundant in the white matter, while 16:0_20:1 shows a homogeneous distribution across the rat brain. For these images, 99th quantile hotspot removal was performed on the non-normalized images.

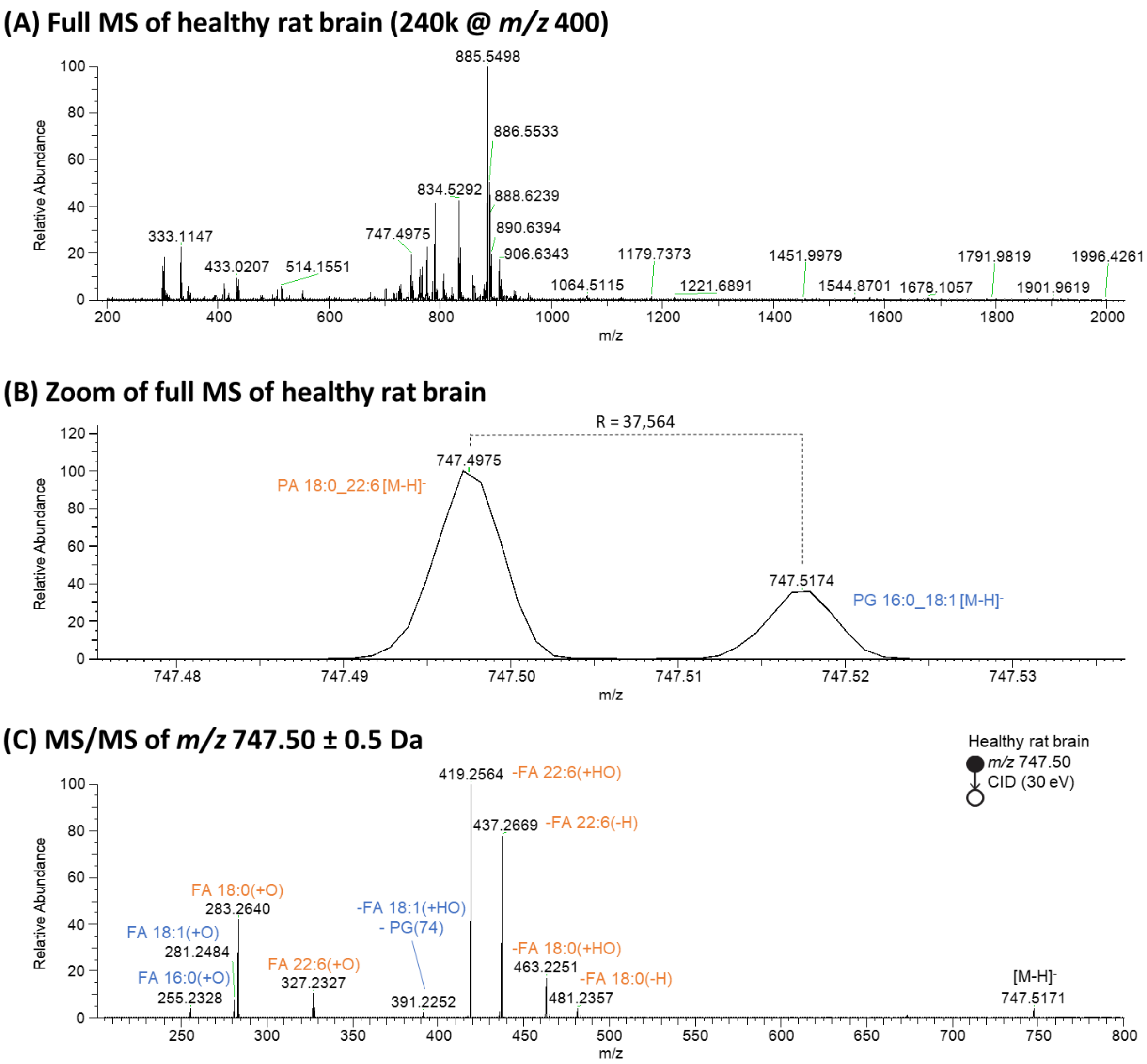

Figure S4: Confirmation of the presence of both PA 40:6 and PG 34:1 in rat brain using Orbitrap and MS/MS. These two lipids differ by only 21 mDa, making them distinguishable in high-resolution mass spectrometers, but not on the SYNAPT platform. (A) A full MS was obtained on a Thermo Orbitrap Elite by averaging 20 scans. Mass resolution was set to 240k (@ *m/z* 400), source pressure was set to 7.5 torr, and the laser was fired at a frequency of 1000 Hz. (B) When zooming in, a clear separation can be observed between the two peaks of PA 40:6 and PG 34:1. (C) MS/MS of *m/z* 747.50 (isolation width 1 Da, 30 eV) confirmed the presence of both PA 40:6 and PG 34:1, allowing for identification as PA 18:0_22:6 and PG 16:0_18:1. This corresponds to the composition found with COzID, where additionally the double bond isomers could be identified as PA 18:0_22:6ω-6, PG 16:0_18:1*n*-7, and PG 16:0_18:1*n*-9.

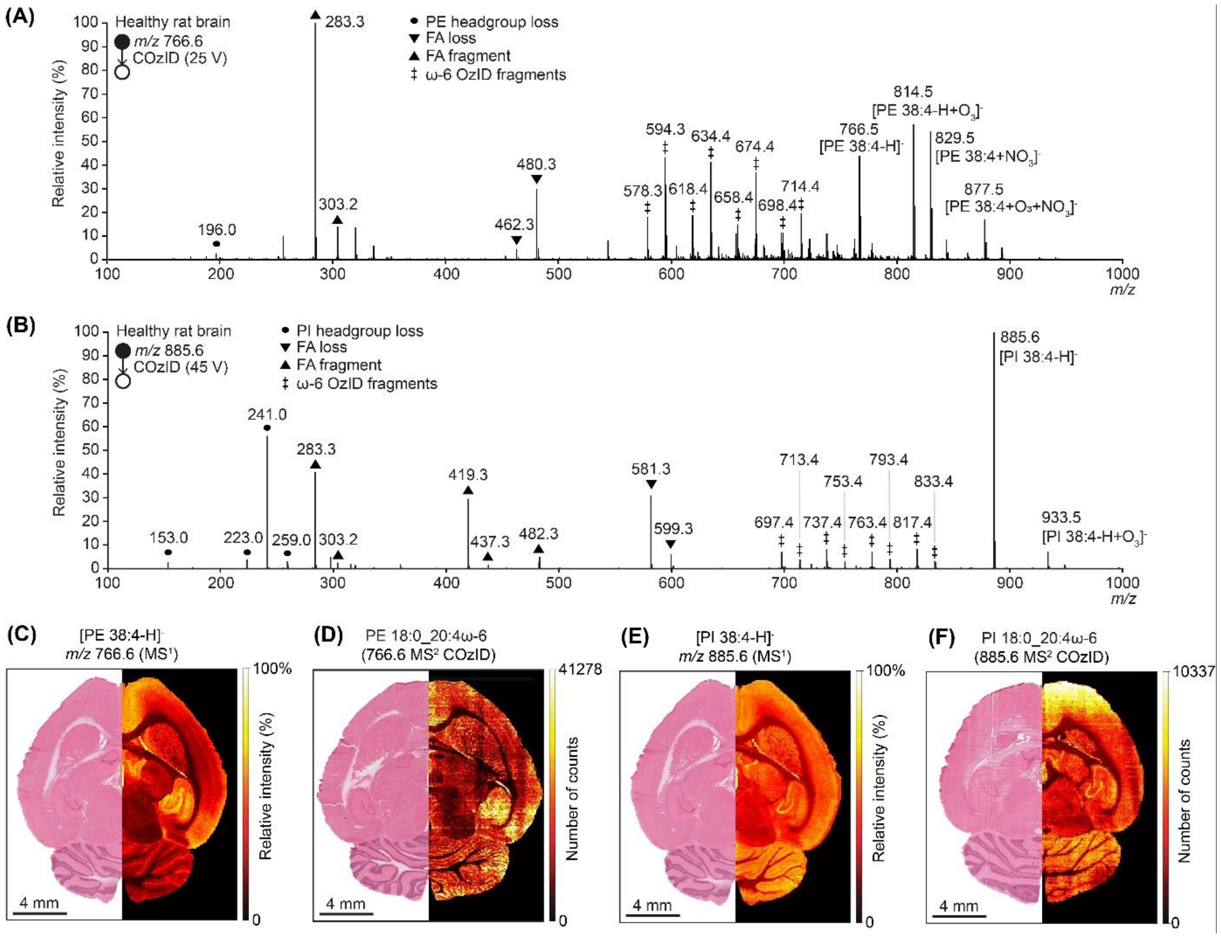

Figure S5: Imaging of [PE 38:4-H]- and [PI 38:4-H]- in rat brain show low isomerization of db positions. (A) Summed spectral average of $[PE\ 38:4\text{-}H]^-$ shows the presence of only PE fragments and ω-6 product ions. (B) Similar to $[PE\ 38:4\text{-}H]^-$, only ω-6 product ions were detected for $[PI\ 38:4\text{-}H]^-$. (C) Image of $[PE\ 38:4\text{-}H]^-$ shows an increase in the dentate gyrus, the ventricle lining, and the molecular layer of the cerebellum. (D) PE 18:0_20:4ω-6 product ions show upregulation similar to the primary ion. (E) Rat brain imaging of $[PI\ 38:4\text{-}H]^-$ shows high abundance of the species in the ventricle lining, the dentate gyrus, and the Purkinje cell layer. (F) Imaging of the summed PI 18:0_20:4ω-6 product ions confirms the same distribution.
